# A guide to ^13^C metabolic flux analysis for the cancer biologist

**DOI:** 10.1038/s12276-018-0060-y

**Published:** 2018-04-16

**Authors:** Maciek R. Antoniewicz

**Affiliations:** Department of Chemical and Biomolecular Engineering, Metabolic Engineering and Systems Biology Laboratory, University of Delaware, Newark, DE 19716 USA

## Abstract

Cancer metabolism is significantly altered from normal cellular metabolism allowing cancer cells to adapt to changing microenvironments and maintain high rates of proliferation. In the past decade, stable-isotope tracing and network analysis have become powerful tools for uncovering metabolic pathways that are differentially activated in cancer cells. In particular, ^13^C metabolic flux analysis (^13^C-MFA) has emerged as the primary technique for quantifying intracellular fluxes in cancer cells. In this review, we provide a practical guide for investigators interested in getting started with ^13^C-MFA. We describe best practices in ^13^C-MFA, highlight potential pitfalls and alternative approaches, and conclude with new developments that can further enhance our understanding of cancer metabolism.

## Cancer research: tagging to understand cancer metabolism

Tracing tagged molecules can help researchers understand the altered metabolism of cancer cells. The abilities of cancer cells to multiply rapidly and invade new tissues are supported by metabolic alterations, which can be investigated by feeding tagged molecules to cells and tracing how they are metabolized. These techniques, such as ^13^C metabolic flux analysis (^13^C-MFA), have been perceived as difficult to use, but recent advances are making them more accessible. Maciek Antoniewicz, University of Delaware, Newark, USA, has published a practical guide for researchers wanting to use 13C-MFA. The review includes best practices, pitfalls, alternative approaches, and new developments, especially new user-friendly software that allows researchers without extensive training in mathematics, statistics, or coding to perform 13C-MFA. Broadening access to tools for investigating altered metabolic pathways may spur development of new cancer therapies targeting these pathways.

## Introduction

In the past decade, measuring intracellular metabolism has become an indispensable tool in biomedical research^1,2^. Cancer metabolism is an especially active area of research^3–8^. It has long been recognized that cancer cells exhibit rewired metabolism compared to normal cells. A century ago, Warburg^9^ described how cancer cells take up large amounts of glucose and preferentially convert it to lactate, even under aerobic conditions. This so-called Warburg effect, or aerobic glycolysis, is a major hallmark of cancer metabolism^10–12^. More recently, with the aid of stable-isotope tracers and network analysis, additional metabolic pathways were identified that are activated in cancer cells, including reductive metabolism of glutamine^13^, altered glycolysis^14^, serine and glycine metabolism^15–17^, one-carbon metabolism^18,19^, transketolase-like 1 (TKTL1) pathway^20,21^, and acetate metabolism^22–25^. The activities of these pathways allow cancer cells to extract cellular building blocks and energy from substrates and use them for cell growth. With the rapid progress in cancer research, an increasingly clearer picture is generated how cancer cells rewire their metabolism, adapt to and manipulate their microenvironment^26–28^, and maintain a continuous supply of anabolic precursors, reducing equivalents and energy to fuel the reproduction of more cancer cells^5,29^.

The complexities of mammalian metabolism require a systems-level analysis of the underlying networks and metabolic phenotypes^30,31^. Currently, ^13^C metabolic flux analysis (^13^C-MFA) is the preferred tool for quantitative characterization of metabolic phenotypes in microbial^32–34^ and mammalian cells^3,4,35–38^. The emergence of ^13^C-MFA as a primary research tool was made possible in large part due to several major advances in theoretical approaches for conducting ^13^C-MFA calculations^39–41^, and more recently, by the availability of dedicated and user-friendly software tools for ^13^C-MFA such as Metran and INCA^42,43^. However, ^13^C-MFA it is still not widely used by cancer biologists, outside of a few expert groups. This may be in part because ^13^C-MFA is sometimes perceived as unintuitive, obscure, demanding in terms of time and data, and costly in terms of initial capital investment and isotopic tracers. Moreover, few guidelines exist to help researchers get started with ^13^C-MFA^44,45^. The main objective of this review is to address these concerns by providing practical guidelines for cancer biologists interested in ^13^C-MFA. First, we describe the basics of ^13^C-MFA, discuss key assumptions that are inherent in ^13^C-MFA but may not always be explicitly stated, highlight best practices in ^13^C-MFA, and identify potential pitfalls as well as alternative approaches. Throughout, we emphasize key aspects that should be considered when planning tracer experiments and performing ^13^C-MFA calculations to ensure correct interpretation of data and results, and to increase insights obtained from these studies.

## Basics of ^13^C-MFA

Cellular metabolism serves four important functions in proliferating cancer cells: (1) supply of anabolic building blocks for cell growth; (2) generation of metabolic energy in the form of ATP to drive thermodynamically unfavorable reactions; (3) generation of redox equivalents in the form of NADPH for anabolic processes such as fatty acid biosynthesis and to combat oxidative stress; and (4) maintaining redox homeostasis by oxidizing excess NADH generated in central metabolic pathways.

**Fig. 1:**
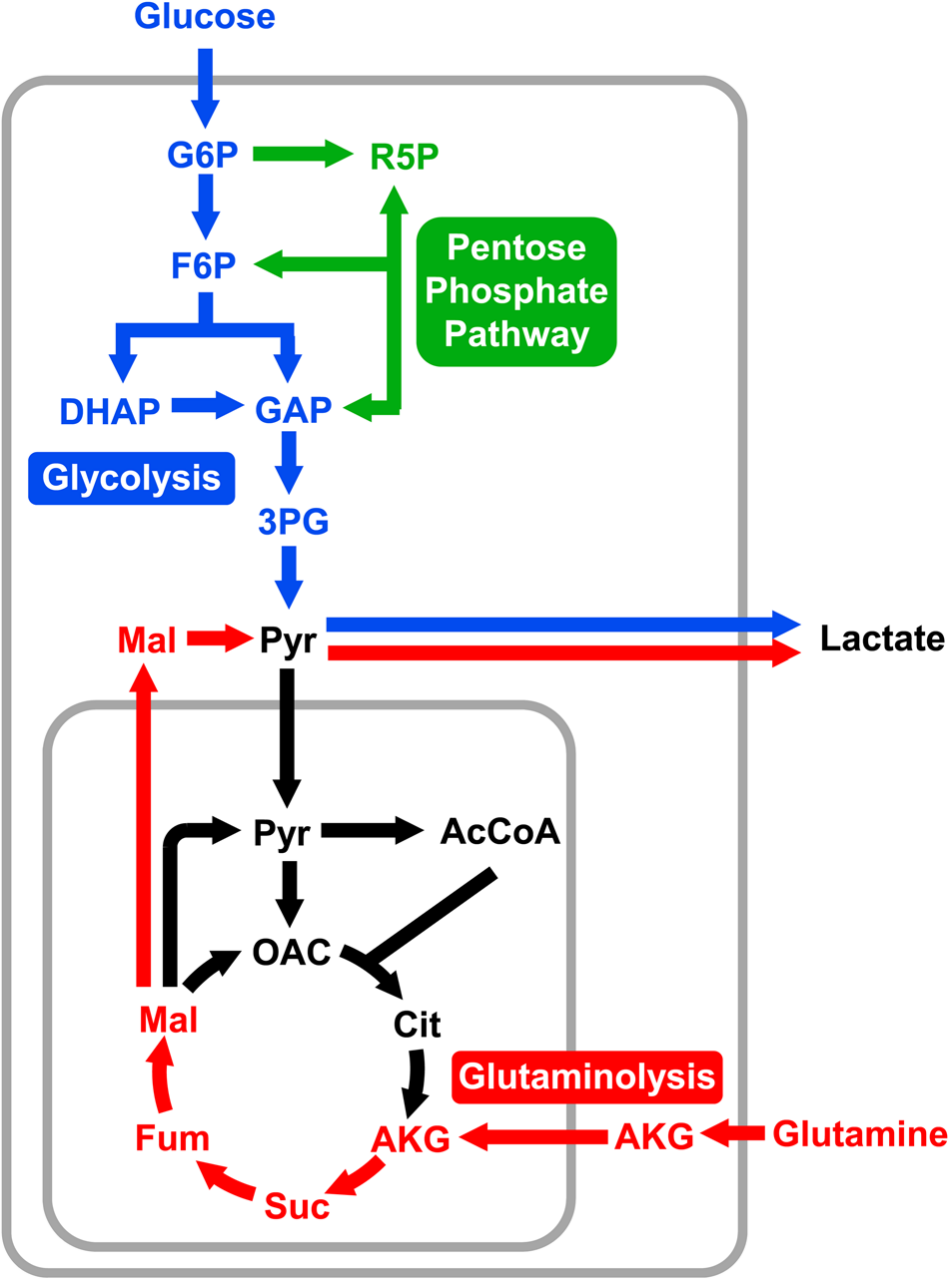
Glucose and glutamine are the two most highly consumed carbon substrates in cancer cells. Both substrates can be converted to lactate via glycolysis and glutaminolysis, respectively. High lactate secretion, especially from glucose, is a major hallmark of cancer cells known as the Warburg effect, or aerobic glycolysis

The first step in obtaining a quantitative picture of cellular metabolism is to measure the growth rate of the cells and quantify nutrient uptake and secretion rates such as glucose and glutamine uptake and lactate secretion^46,47^ (Fig. 1). These external rates provide important boundary constraints on intracellular pathway activities. However, due to redundancies in mammalian metabolic pathways, external rates alone do not allow detailed conclusions to be drawn about the relative contribution of specific metabolic pathways to overall metabolism^46,48^. To examine intracellular fluxes in detail, stable isotopes such as ^13^C are utilized. When a labeled substrate, e.g., [1,2-^13^C]glucose, is metabolized by cells, enzymatic reactions rearrange carbon atoms resulting in specific labeling patterns in downstream metabolites that can be measured with analytical techniques such as mass spectrometry (MS), or nuclear magnetic resonance. For a well-selected tracer, different metabolic pathways will produce distinctly different labeling patterns in the measured metabolites from which fluxes can be inferred^49,50^. However, in most cases, isotopic labeling data cannot be interpreted intuitively due to the highly complex nature of atom rearrangements in metabolic pathways^51^; instead, a formal model-based analysis approach is required to extract flux information from the labeling data. In the past 20 years, ^13^C-MFA has emerged as the primary approach used for converting isotopic labeling data into corresponding metabolic flux maps^45^.

**Fig. 2:**
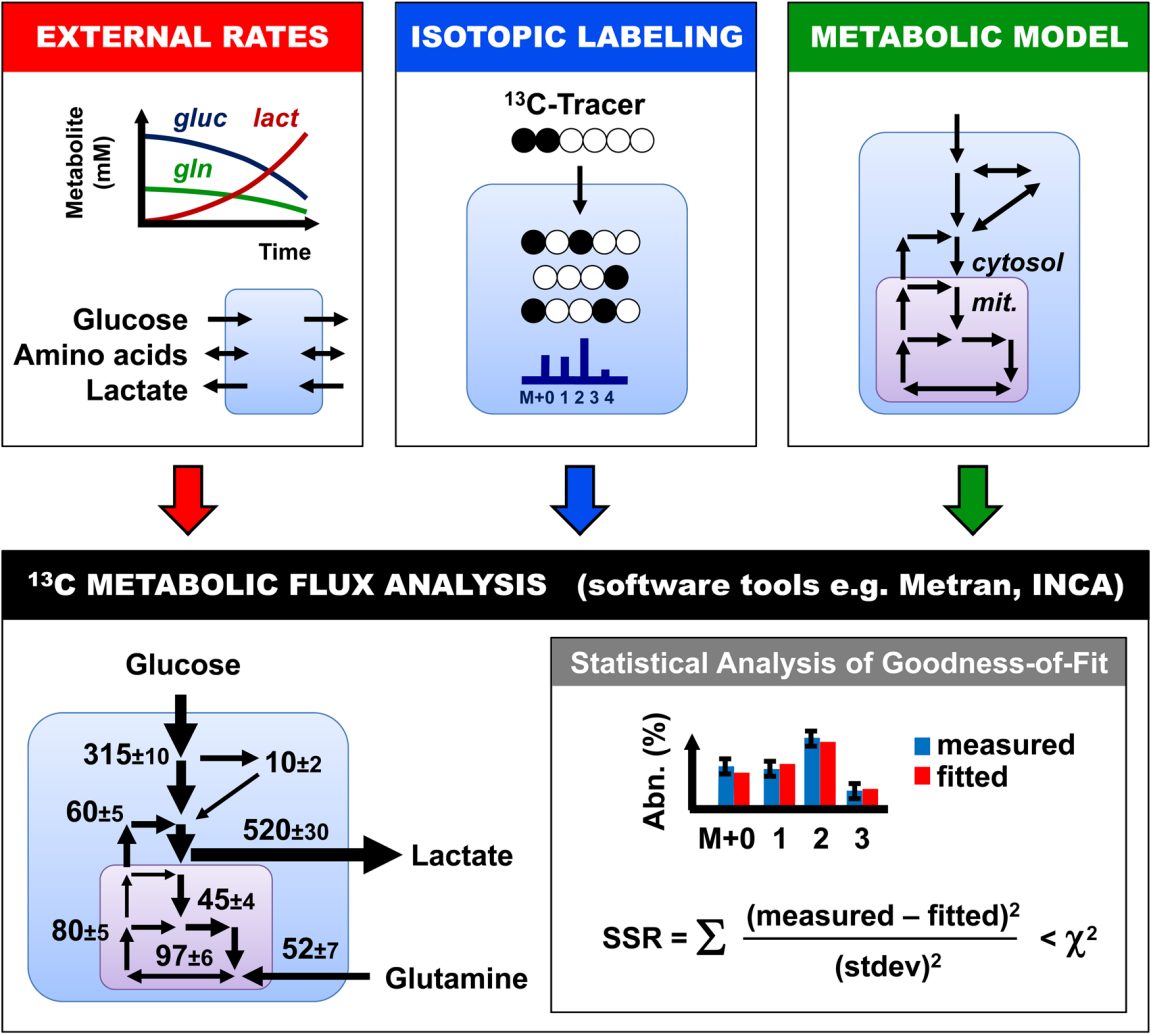
^13^C metabolic flux analysis (^13^C-MFA) is a powerful approach for quantifying intracellular metabolic fluxes in cancer cells. The three inputs required for ^13^C-MFA are external uptake and secretion rates, isotopic labeling measurements, and a comprehensive compartmentalized model of cellular metabolism. User-friendly software tools for ^13^C-MFA, such as Metran and INCA, can be used to perform ^13^C-MFA calculations. These tools produce as outputs fluxes for all reactions in the model, confidence intervals for the estimated fluxes, and statistical analysis of the goodness-of-fit

The main objective of ^13^C-MFA is thus to generate a quantitative map of cellular metabolism by assigning flux values to the reactions in the network model and confidence intervals for each estimated flux (Fig. 2). At a high level, ^13^C-MFA is formulated as a least-squares parameter estimation problem, where fluxes are unknown model parameters that must be estimated by minimizing the difference between the measured labeling data and labeling patterns simulated by the model, subject to stoichiometric constraints resulting from mass balances for intracellular metabolites and metabolite labeling states, the so-called isotopomers^40,52^. When ^13^C-MFA first emerged in 1990s^53^, the main challenge was to develop efficient algorithms for solving large sets of isotopomer mass balances^54^. Eventually, the computational problems in ^13^C-MFA were resolved with the development of the elementary metabolite unit (EMU) framework that allows efficient simulation of isotopic labeling in any arbitrary biochemical network model^39^. The EMU framework was subsequently incorporated into user-friendly software tools for ^13^C-MFA, such as Metran and INCA^42,43^, that are freely available to the scientific community. These powerful tools have opened up ^13^C-MFA to a much wider scientific audience, including cancer biologists, that may not have extensive background in mathematics and statistics, which was required before these software packages became available. In the next sections, we describe in detail the three inputs that are required for performing ^13^C-MFA calculations: (i) external rates; (ii) isotopic labeling; and (iii) metabolic model (Fig. 2).

## Determination of external rates

To quantify intracellular metabolic fluxes, first, the cross talk between the cells and their environment must be quantified. Collectively referred to as external rates, this includes measuring influxes of nutrients such as glucose and glutamine, and secretion of metabolic by-products such as lactate and glutamate. In addition, the rate of cell growth must be determined. Assuming cells are continuously dividing, the cell number will increase exponentially according to:

1 $$N_x = N_{x,0} \cdot {\rm{exp}}(\mu \cdot t)$$

Here *N*_*x*_ is the number of cells (typically expressed in millions of cells), and *µ* (1/h) is the growth rate. The growth rate is easily determined by plotting the natural logarithm of *N*_*x*_ vs time and determining the slope of the curve. If cells are counted only at two time points, then the growth rate is determined as follows:

2 $$\mu = \frac{{\ln \left( {N_{x,t2}} \right) - \ln \left( {N_{x,t1}} \right)}}{{\Delta t}}$$

The doubling time (*t*_d_) is inversely related to the growth rate, according to:

3 $$t_{\mathrm{d}} = \ln \left( 2 \right)/\mu$$

External rates, i.e., nutrient uptake rates and waste product secretion rates, can be determined in a straightforward way by measuring changes in metabolite concentrations during the labeling experiment. For exponentially growing cells, external rates (*r*_*i*_, in units nmol/10^6^ cells/h) can be calculated as follows:

4 $$r_i = 1000 \cdot \frac{{\mu \cdot V \cdot \Delta C_i}}{{\Delta N_x}}$$

Here Δ*C*_*i*_ (mmol/L) is the change in concentration of a particular metabolite *i* between two sampling time points, Δ*N*_*x*_ is the change in cell number (expressed in millions of cells) during the same time period, *V* (mL) is the culture volume, and *µ* (1/h) is the growth rate. Based on this expression, external rates have negative values for uptake rates and positive values for secretion rates. For non-proliferating cells, external rates are determined by a slightly different expression:

5 $$r_i = 1000 \cdot \frac{{V \cdot \Delta C_i}}{{\Delta t \cdot N_x}}$$

Because glutamine is an unstable molecule, i.e., it spontaneously degrades to pyroglutamate and ammonium under normal culture conditions, the calculated glutamine uptake rate must be corrected for glutamine degradation, i.e., the measured rate reflects both net uptake of glutamine by the cells and glutamine degradation. Glutamine degradation can be expressed as a first-order degradation process with a degradation constant of around 0.003/h^55^. After correcting for glutamine degradation^55^, the true net glutamine uptake rate is obtained. For long tracer experiments (e.g., >24 h), it may also be necessary to correct for evaporation effects. For this purpose, control experiments without cells are performed. By measuring the apparent increases in metabolite concentrations over time, the rate of evaporation can be estimated. The dynamics of glutamine degradation are also easily determined from these control experiments.

For ^13^C-MFA studies, external rates are often determined for glucose uptake, lactate secretion, and amino-acid uptake and secretion. For proliferating cancer cells, typical values are as follows: 100–400 nmol/10^6^ cells/h for glucose uptake; 200–700 nmol/10^6^ cells/h for lactate secretion; 30–100 nmol/10^6^ cells/h for glutamine uptake; and 2–10 nmol/10^6^ cells/h for uptake or secretion of other amino acids. Depending on the scope of the study, it may also be important to measure the rates of other metabolites such as ammonium, pyruvate, acetate, citrate, and any other significant nutrients or by-products that cancer cells exchange with their environment.

## Measurement of isotopic labeling

**Fig. 3:**
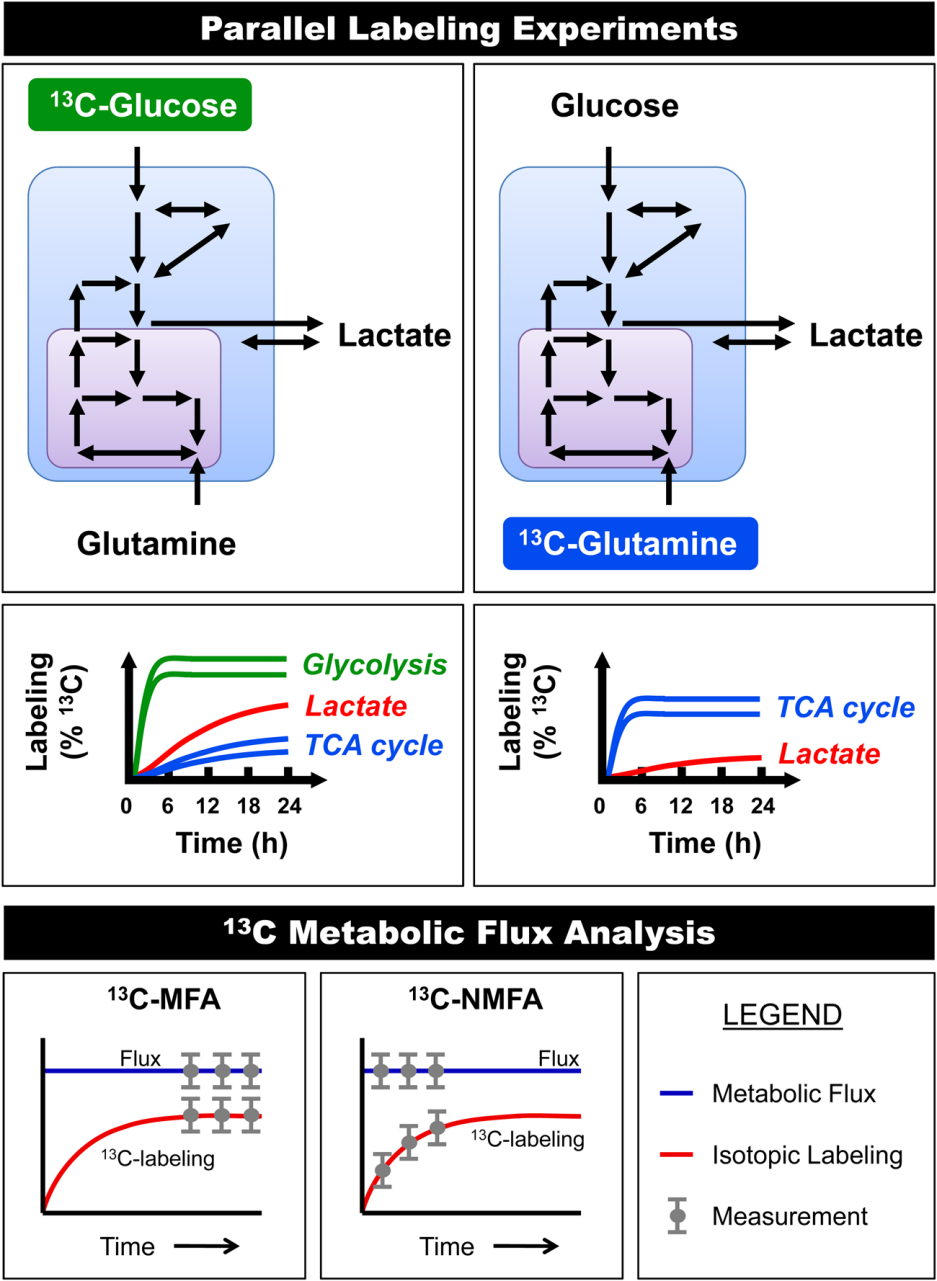
Parallel labeling experiments with different ^13^C-labeled substrates greatly enhance the resolution of metabolic fluxes in complex models. The rate of labeling incorporation after the introduction of a ^13^C-tracer depends on the turnover rate of intracellular metabolites and exchanges between intracellular and extracellular metabolites. In particular, external lactate can slow down labeling of intracellular pyruvate and TCA cycle metabolites from ^13^C-glucose tracers. If isotopic steady state is reached then labeling data can be analyzed with ^13^C-MFA. However, if the system has not reached isotopic steady state, then the labeling data must be analyzed using isotopic non-stationary ^13^C-MFA (^13^C-NMFA)

When conducting ^13^C-tracer experiments, a labeled substrate is introduced to the culture medium that is then taken up by the cells and metabolized through various metabolic pathways. It takes a certain amount of time before intracellular metabolites reach a constant labeling state, which is referred to as isotopic steady state^46^ (Fig. 3). The time required to reach isotopic steady state depends on the turnover rate of metabolites in a pathway and the labeling dynamics of upstream metabolites that feed into the pathway. The turnover rate of a metabolite pool is roughly equivalent to the ratio of the metabolite pool size and the flux through that metabolite pool. For proliferating cells, isotopic steady state can be reached relatively quickly, i.e., within a few hours after the introduction of the isotopic tracer^56^. However, in some cases, due to exchange of intracellular and external metabolites, significantly slower labeling incorporation rates can be observed. In particular, external lactate often acts as a large buffer that slows down labeling of intracellular pyruvate and downstream metabolic pathways, e.g., tricarboxylic acid (TCA) cycle, when ^13^C-glucose tracers are used^55^. Slow labeling may be observed even if there is large net secretion of lactate, since external lactate readily exchanges with intracellular lactate, which in turn rapidly equilibrates with cytosolic pyruvate. The effective pool size of intracellular pyruvate thus becomes the combined pool of intracellular pyruvate, intracellular lactate, and external lactate. This buffering effect can be so extreme that certain metabolites may never reach isotopic steady state^55^. One strategy to reduce the buffering effect of lactate is to ensure that little or no lactate is present in the medium at the beginning of ^13^C-glucose tracer experiments.

An important inherent assumption of ^13^C-MFA calculations is that all metabolites are at isotopic steady state. It is thus critical to validate this assumption for all tracer experiments performed. To validate this, isotopic labeling is measured for at least two time points, e.g., 18 and 24 h, after the introduction of tracer. If isotopic labeling is identical for the two time points, then isotopic steady state is confirmed and the labeling data can be analyzed using classical ^13^C-MFA. However, if isotopic labeling is changing with time, then the data must be analyzed using a more advanced ^13^C-MFA approach called isotopic-non-stationary ^13^C-MFA, or ^13^C-NMFA^41^. Most software packages for ^13^C-MFA can only perform classical ^13^C-MFA calculations, i.e., assuming isotopic steady state, although a few software packages such as INCA can perform both ^13^C-MFA and ^13^C-NMFA calculations^43^.

MS is currently the preferred analytical technique used for measuring isotopic labeling of intracellular metabolites. With the technological advances in gas chromatography/MS (GC/MS) and liquid chromatography/MS (LC/MS) in the past two decades, it is now possible to measure mass isotopomer distributions for a large number of intracellular metabolites from as few as one million cells, including for intermediates of glycolysis pathway: fructose 6-phosphate (F6P), dihydroxyacetone phosphate, glycerol 3-phosphate, 3-phosphoglycerate (3PG), phosphoenolpyruvate, pyruvate, and lactate; intermediates of the pentose phosphate pathway (PPP; LC/MS mainly): xylulose 5-phosphate (X5P), ribose 5-phosphate (R5P), and sedoheptulose 7-phosphate; intermediates of the TCA cycle: citrate, α-ketoglutarate (AKG), succinate, fumarate, and malate; and most amino acids, including alanine, aspartate, glutamate, glutamine, proline, serine, and glycine.

## Parallel labeling experiments

The selection of an isotopic tracer (or multiple tracers) is one of the most important considerations when designing ^13^C-MFA studies, since this ultimately determines the quality (i.e., precision and accuracy) of flux results that can be obtained^50^. It is now well-known that there is no single best tracer for ^13^C-MFA studies. Generally, ^13^C-glucose tracers are best for determining fluxes in upper metabolism (e.g., glycolysis and PPP), while ^13^C-glutamine tracers typically produce better resolution of fluxes in lower parts of metabolism (e.g., TCA cycle and reductive carboxylation)^57,58^ (Fig. 3). A powerful approach to achieve high resolution of multiple metabolic pathways is to perform parallel labeling experiments with different tracers and then integrate all data into a single comprehensive flux model^59,60^. For example, parallel labeling experiments with [1,2-^13^C]glucose and [U-^13^C]glutamine have been demonstrated to be particularly informative and complementary^56,58,61^. When conducting parallel labeling experiments, it is important that the only difference between the experiments is which metabolite is labeled, i.e., concentrations of all nutrients in the media must be the same for parallel labeling experiments^62^. With recent advances in ^13^C-MFA methodology it is now fairly straightforward to analyze isotopic labeling data from parallel labeling experiments^45^. The Metran software was the first tool that allowed comprehensive analysis of parallel labeling experiments for high-resolution ^13^C-MFA. Recently, other ^13^C-MFA software packages have also included this feature.

## Metabolic model for ^13^C-MFA

All ^13^C-MFA calculations are based on a model of biochemical reactions within a specified metabolic network. Determining the scope of the model is an important decision in ^13^C-MFA studies. Unfortunately, there is only limited consensus in the literature on the optimal scope of metabolic models for flux analysis in cancer cells. This is in part due to the fact that the appropriate model complexity will depend to some degree on the specific choice of isotopic tracer (or tracers), how many parallel labeling experiments are performed, and how many and which labeling measurements are collected. In general, more comprehensive data sets, i.e., based on multiple parallel labeling experiments with different labeled substrates^36,56,60,63^, will permit the use of more complex models for ^13^C-MFA than smaller data sets obtained using a single tracer experiment.

**Fig. 4:**
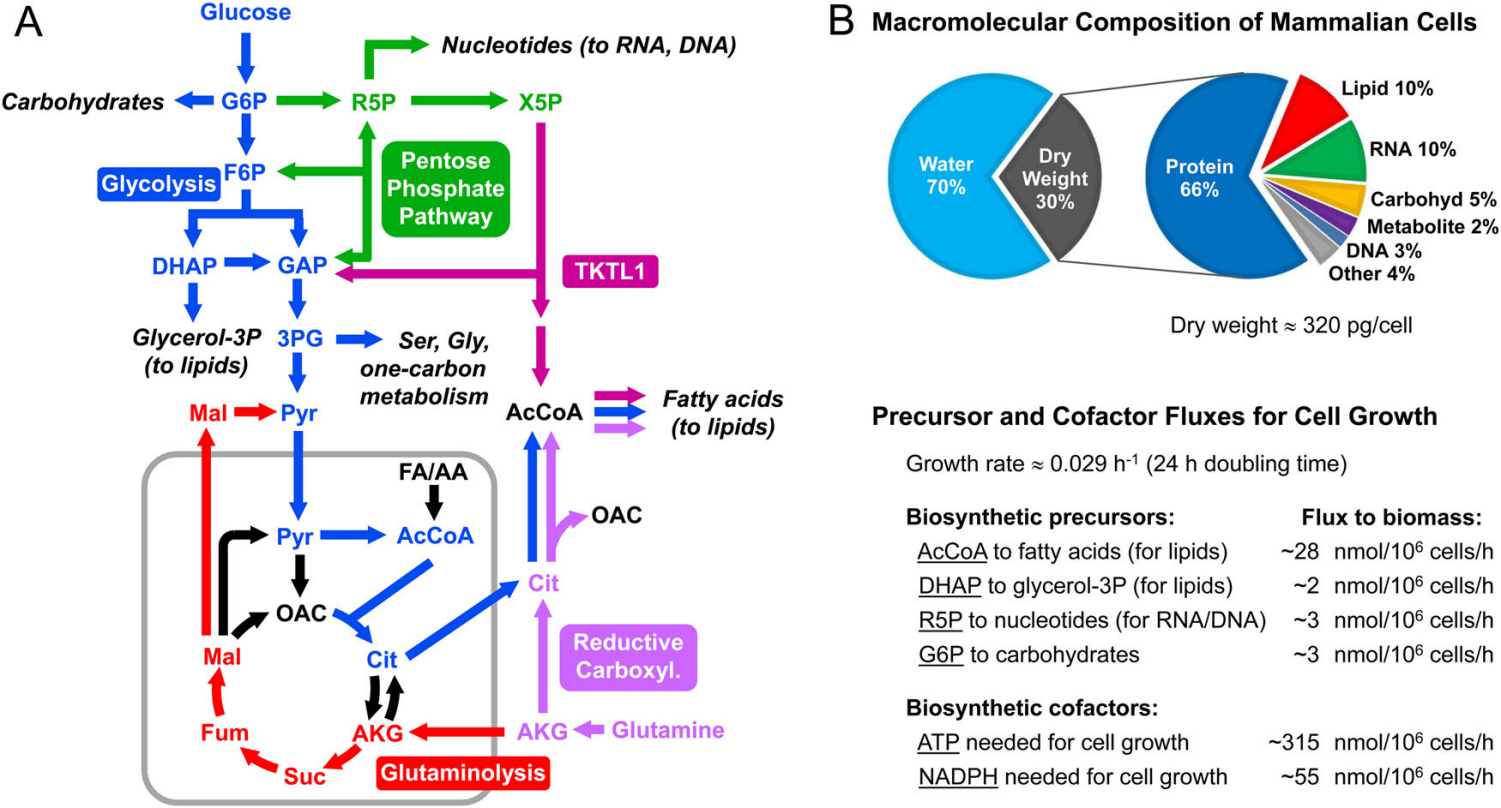
^13^C metabolic fluxes are estimated based on comprehensive compartmentalized models of cellular metabolism. **a** The diagram shows important metabolic pathways in cancer metabolism, including glycolysis, pentose phosphate pathway, TCA cycle, reductive carboxylation of glutamine, and transketolase-like 1 (TKTL1) pathway. One of the key functions of cellular metabolism is to supply anabolic building blocks needed for cell growth, shown here as draining reactions from central metabolic pathways. **b** A typical macromolecular composition of cancer cells is shown. The macromolecular composition and the growth rate of cells determine the rates at which anabolic precursors must be produced to sustain cell growth. Typical values of anabolic precursor fluxes in proliferating cancer cells are shown

Typically, ^13^C-MFA models will include all major metabolic pathways of central carbon metabolism such as glycolysis, PPP, TCA cycle, as well as any relevant reactions that connect these pathways (Fig. 4a). Compartmentalization of metabolites and metabolic reactions is an important feature of mammalian cells that must be captured in the model. Metabolites and reactions are therefore assigned to specific metabolic compartments such as cytosol or mitochondrion. Certain metabolites will be present in multiple compartments, for example, pyruvate, acetyl coenzyme A, citrate, malate, fumarate, oxaloacetate, and AKG. These metabolites are treated as separate entities in the model that can have different labeling states in different compartments. Transport reactions in the model allow specific metabolites to be transferred between cellular compartments. Compartment-specific isozymes, which can operate independently, must be included as separate reactions in the model (e.g., cytosolic and mitochondrial isocitrate dehydrogenases; and cytosolic and mitochondrial malic enzymes). Finally, ^13^C-MFA models will include a lumped biomass formation reaction that drains anabolic precursors from central metabolism (and extracellular medium, e.g., essential amino acids) for the biosynthesis cellular macromolecules^55^. The stoichiometric coefficients for this lumped biomass reaction are easily determined based on the macromolecular composition of cells (Fig. 4b). Recently, a number of GC/MS-based protocols have been developed that allow biomass compositions of cells to be determined easily and accurately^64–66^. Typical values for anabolic precursor effluxes for proliferating cancer cells are shown in Fig. 4b.

## ^13^C-MFA and statistical analysis

Current software tools for ^13^C-MFA such as Metran and INCA are designed so that users are not required to have any extensive background in mathematics, statistics, or writing computer code. All of the complex math associated with performing ^13^C-MFA computations is hidden from the user. These software tools accept as inputs: (1) a user-defined metabolic network model consisting of biochemical reactions and corresponding atom transitions; and (2) a set of measurements consisting of isotopic labeling data and external rates. As outputs, the software returns the following: (1) metabolic fluxes for the entire network; (2) confidence intervals for all estimated fluxes; and (3) statistical analysis of the goodness-of-fit (Fig. 2).

^13^C-MFA should be viewed as an iterative process that requires careful scrutiny of the analysis results. After the software returns a result, it is up to the user to determine how acceptable the result is, and this requires some level of experience. Generally, it is rare that the first result returned by the software will be the optimal solution. There are several important reasons for this. First, as mentioned in the introduction, in ^13^C-MFA a highly nonlinear multi-dimensional parameter estimation problem is solved^40^. Problems of this kind have many suboptimal local solutions, and there is no guarantee that the first solution returned by the software will be the global optimal solution. To address this concern, ^13^C-MFA is typically restarted many times with random initial values for all fluxes and the goodness-of-fit of these iterations is compared. The goodness-of-fit is expressed by the sum of squared weighted residuals, or the SSR value^40^ (Fig. 2). The lower the SSR value, the better the agreement between the measured data and the model fit. Assuming that the metabolic model is correct and data are without gross measurement errors, the minimized SSR is a stochastic variable with a *χ*^2^-distribution. Based on this property, it is possible to calculate a maximum statistically acceptable value for SSR, which is roughly equal to the number of fitted measurements (*n*) minus the number of estimated independent parameters (*p*). More technically, the acceptable range of SSR values is between *χ*^2^_*α*/2_(*n* − *p*) and *χ*^2^_1−*α*/2_(*n* − *p*), where *α* is a certain chosen threshold value, for example, 0.05 for the 95% confidence interval.

The strategy for performing ^13^C-MFA is thus to restart flux estimation many times (typically at least 10 times, but more is preferred) and compare the SSR values. The solution with the lowest SSR value is then selected as the optimal solution. Often, multiple iterations will produce the same low SSR value, which increases the likelihood that the solution is indeed the global optimal solution. In practice, however, it is not uncommon that the lowest SSR value obtained in this way is still greater than the maximum statistically allowed SSR. Some common reasons for this are as follows:

1. Errors in the metabolic model. Mistakes in the user-specified metabolic model such as incorrect reaction stoichiometries or errors in atom transitions are generally easy to identify and correct.
2. Incomplete metabolic model. Omitting important reactions or pathways from the model will result in poor fits. Thus, depending on the quality of fit, the scope of the model may need to be adjusted. In some cases, it may be necessary to include hypothetical reactions in the model in order to achieve an acceptable fit. In this way, ^13^C-MFA can be used as a hypothesis generating tool that can eventually lead to the discovery of novel metabolic pathways or reactions^67–72^. As an example, the TKTL1 pathway was recently discovered in Chinese hamster ovary cells by this approach^73^.
3. Gross measurement errors. It is not uncommon that certain labeling data will contain gross measurement errors, for example, due to co-elution of metabolites in GC/MS and LC/MS analyses. Careful inspection of ion chromatograms can in most cases help to identify co-elution problems. In such cases, labeling data for the contaminated metabolite fragments should be excluded from flux analysis.
4. Incorrect assumptions about measurement errors. The SSR value is calculated by summing up the weighted squared differences between the measured and simulated values. The weighting factors are inverses of measurement standard deviations squared. The assumed measurement errors thus greatly influence the calculated SSR value. Typical measurement errors used in ^13^C-MFA studies are as follows: 0.004 (or 0.4 mol%) for GC/MS data; 0.01 (or 1 mol%) for LC/MS data; and 5–10% relative error for external rates. In cases when very high or very low SSR values are obtained, it may be necessary to reevaluate the assumptions regarding measurement errors. Moreover, inspection of weighted residuals can inform if correct measurement errors have been assigned. Assuming measurement errors are random, the weighted residuals should follow a normal distribution *N*(*µ* = 0, *σ*^2^ = 1), which can be easily tested^40^.

## Isotopomer spectral analysis

**Fig. 5:**
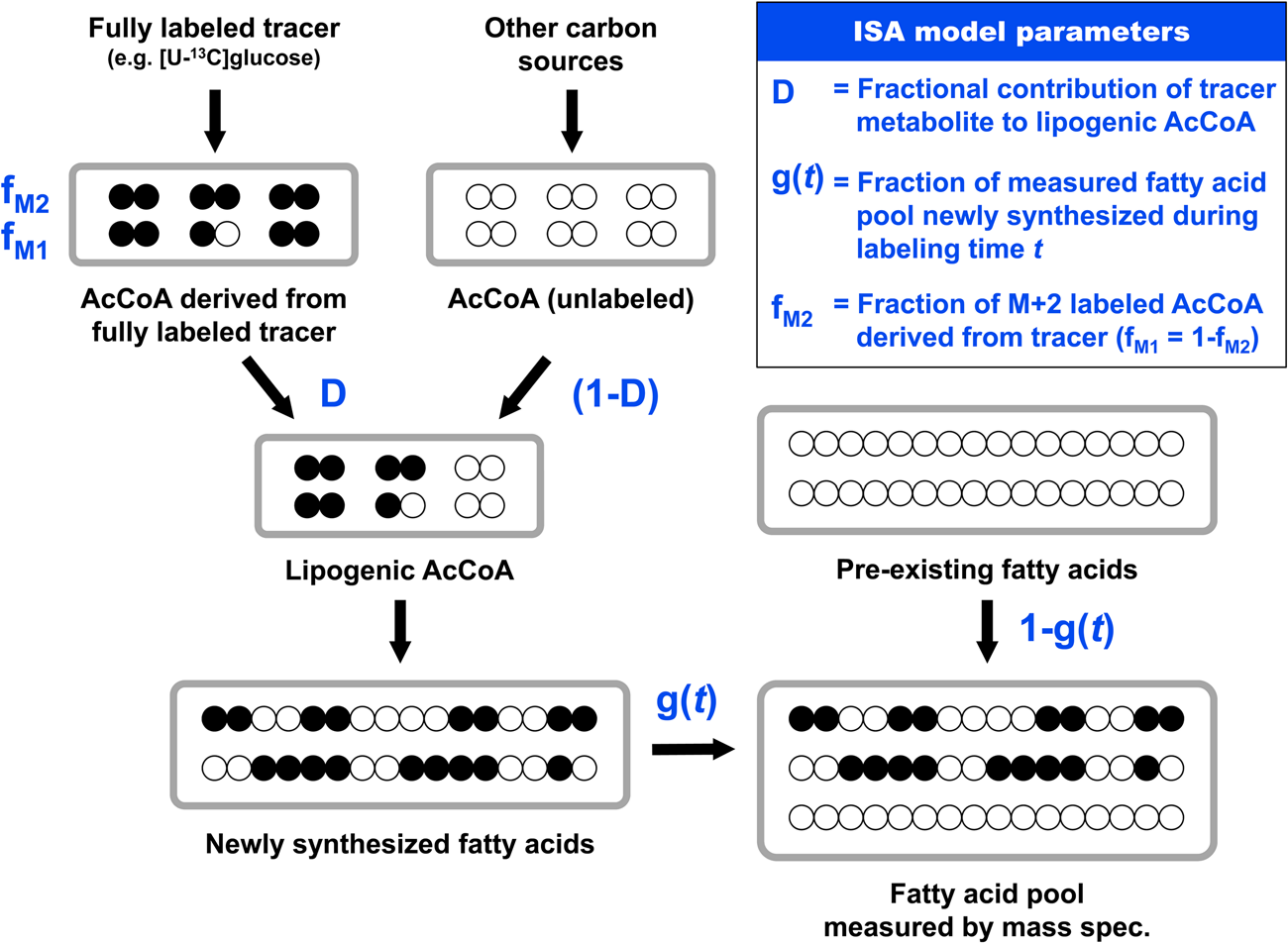
The isotopomer spectral analysis (ISA) approach is used to quantify de novo fatty acid biosynthesis based on tracer experiments with fully ^13^C-labeled substrates. In the classical ISA formulation, two model parameters are determined, the *D*-value and the *g*(*t*)-value. The ISA approach can be generalized and extended to include additional model parameters such as f_M2_

Isotopomer spectral analysis (ISA) is a related and widely used analysis approach for analyzing de novo fatty acid biosynthesis^74^ (Fig. 5). ISA calculations can be performed with most current software tools for ^13^C-MFA. Initially developed in early 1990s (before the ^13^C-MFA approach was fully formalized), the ISA approach is based on a relatively simple two-parameter model for analyzing mass isotopomer distributions of fatty acids from tracer experiments with fully ^13^C-labeled substrates, e.g., [U-^13^C]glucose. In the classical ISA formulation, two model parameters are determined: the *D*-value and the *g*(*t*)-value^74^. The *D*-value quantifies the fractional contribution of the fully ^13^C-labeled metabolite to lipogenic AcCoA, and the *g*(*t*)-value quantifies the fraction of fatty acids that were newly synthesized during the labeling time *t*.

Typically, several parallel labeling experiments are performed with different fully ^13^C-labeled substrates, e.g., [U-^13^C]glucose and [U-^13^C]glutamine, and isotopic labeling is measured for multiple fatty acids is each experiment, e.g., C16:0, C16:1, C18:0, and C18:1, using GC/MS. In theory, for a given tracer the *D*-values should be identical for all fatty acids, since all fatty acids are derived from the same cytosolic AcCoA pool. In contrast, the *g*(*t*)-values may be different for each fatty acid since different fatty acids may be synthesized at different rates. However, *g*(*t*)-values for a particular fatty acid determined with different tracers, e.g., with [U-^13^C]glucose and [U-^13^C]glutamine, should be the same since the synthesis rate of a particular fatty acid should not depend on which substrate is labeled. The ISA approach can be generalized for analysis of odd-chain fatty acids, e.g., C15:0 and C17:0, as was recently demonstrated^62^. Moreover, ISA can be extended to include additional model parameters^62^ (Fig. 5). In the classical ISA model, it is assumed that fully labeled substrates, e.g., [U-^13^C]glutamine, will produce only fully labeled AcCoA (i.e., M + 2-labeled). However, this assumption may not always be valid. For example, metabolism of [U-^13^C]glutamine in the TCA cycle can result in some loss of ^13^C, which will produce a mixture of M + 1- and M + 2-labeled AcCoA. Moreover, catabolism of certain substrates such as [U-^13^C]leucine will always produce a mixture of M + 1- and M + 2-labeled AcCoA due to carbon exchange with unlabeled CO_2_^75^. For example, for the case of [U-^13^C]leucine, 33% of AcCoA will be M + 1-labeled and 67% of AcCoA will be M + 2-labeled^62^. By including an additional f_M2_ parameter in the ISA model, losses of ^13^C atoms can be captured, which produces more accurate estimates of *D*- and *g*(*t*)-values.

As indicated above, ISA analysis is typically performed with different fully ^13^C-labeled substrates in parallel experiments. These studies provide important insights into the relative contributions of different nutrients for de novo lipogenesis^13,76^. The estimated *g*(*t*)-values are also informative, since they can be used to calculate absolute de novo biosynthesis rates of fatty acids (nmol/10^6^ cells/h):

6 $${\rm{Fatty}}\,{\rm{acid}}\,{\rm{biosynthesis}}\,{\rm{rate}} = \frac{{{\rm{FA}}}}{{{\rm{\Delta }}t}} \cdot \frac{{g(t)}}{{1 - g\left( t \right)}}$$

Here FA is the macromolecular content of a particular fatty acid in cancer cells (in units nmol/10^6^ cells; a typical value for palmitate is 40 nmol/10^6^ cells), and Δ*t* (h) is the length of the tracer experiment. The fatty acid content of cancer cells is easily determined with GC-flame ionization detector, or using the protocols described by Long and Antoniewicz^65^.

## Quantifying fluxes in upper metabolism

In the next two sections, we describe briefly common stable-isotope tracing strategies for determining fluxes in upper and lower parts of central carbon metabolism, respectively. When performing flux analysis in upper metabolism, the drain of metabolic precursors toward biomass synthesis such as glucose 6-phosphate (G6P) for carbohydrates, R5P for nucleotides, and glycerol 3-phosphate for lipids can be generally ignored, since the glucose uptake rate (~100–400 nmol/10^6^ cells/h) is typically two orders of magnitude greater than the drain of anabolic precursors for cell growth (~2–3 nmol/10^6^ cells/h; Fig. 4). However, when performing flux analysis in lower metabolism, the drain of AcCoA for lipogenesis (~28 nmol/10^6^ cells/h) cannot be ignored since this flux is comparable in magnitude to other fluxes in lower metabolism.

**Fig. 6:**
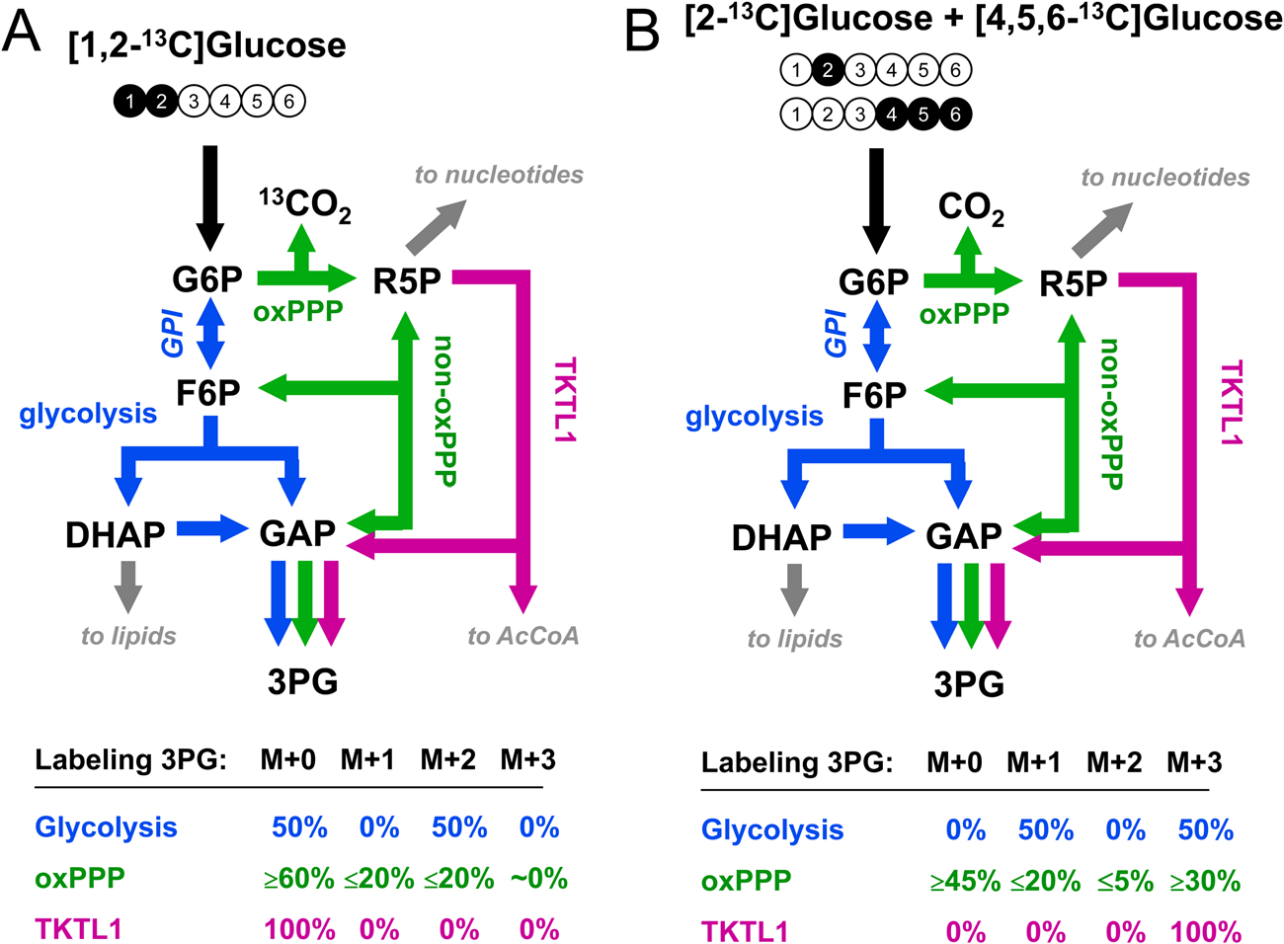
Two alternative ^13^C-glucose-tracing strategies for analysis of metabolic fluxes in upper metabolism based on mass isotopomer measurements of 3-phosphoglycerate (3PG). **a** The [1,2-^13^C]glucose tracer allows good resolution of relative glycolysis and pentose phosphate pathway fluxes. **b** A mixture of 50% [2-^13^C]glucose and 50% [4,5,6-^13^C]glucose is an improved tracer approach that also allows precise quantification of the transketolase-like 1 (TKTL1) pathway flux

At present, [1,2-^13^C]glucose is one of the most widely used tracers to quantify fluxes of glycolysis and PPP (Fig. 6a). With this tracer the two pathways produce distinctly different labeling patterns in downstream metabolites such as 3PG, which can be easily measured with GC/MS and LC/MS. Metabolism of glucose via glycolysis produces 3PG that is 50% M + 2-labeled and 50% unlabeled (i.e., M + 0), while metabolism of glucose via oxidative PPP (oxPPP) produces a mixture of M + 0-, M + 1-, and M + 2-labeled 3PG. For a single pass through oxPPP, the labeling of 3PG is 60% M + 0, 20% M + 1, and 20% M + 2. The ratio of M + 1/M + 2 mass isotopomers of 3PG thus roughly approximates the relative contribution of oxPPP to glucose metabolism. However, this approximation should be used with caution. Specifically, the reversible G6P isomerase reaction, which interconverts G6P and F6P, can reroute a significant fraction of F6P that is produced via PPP back to G6P to be metabolized via oxPPP a second time (and possibly a third time), which results in additional losses of ^13^C (Fig. 6a). Thus, depending on the equilibration of F6P and G6P, the M + 1 and M + 2 mass isotopomers of 3PG can be significantly <20% and the ratio M + 1/M + 2 may be different from unity. Thus, to obtain a reliable estimate of oxPPP flux, the 3PG labeling data should be analyzed formally with ^13^C-MFA.

Recently, a third metabolic pathway was discovered in cancer cells by which glucose can be metabolized, the TKTL1 pathway, which converts X5P (an intermediate of PPP) to glyceraldehyde 3-phosphate and a two-carbon metabolite, likely acetate, which can be further metabolized to cytosolic AcCoA^20,21^ (Fig. 6). Unfortunately, [1,2-^13^C]glucose and several other commonly used glucose tracers cannot provide a reliable estimate of the TKTL1 flux. To address this limitation, alternative glucose-tracing strategies have been developed to better resolve the three glucose metabolism pathways, glycolysis, PPP, and TKTL1^73^. One of the best tracer strategies was based on mixtures of 50% [4,5,6-^13^C]glucose and 50% of either [1-^13^C]glucose, [2-^13^C]glucose, or [3-^13^C]glucose (Fig. 6b). With these tracers, it is possible to determine precise fluxes of all three metabolic pathways, as recently demonstrated in Chinese hamster ovary cells^73^. Other optimal glucose tracers have also been proposed for analysis of specific metabolic pathways; for example, [3,4-^13^C]glucose was determined to be a particularly good tracer for quantifying the anaplerotic flux of glucose into the TCA cycle^57,77,78^.

## Quantifying fluxes in lower metabolism

**Fig. 7:**
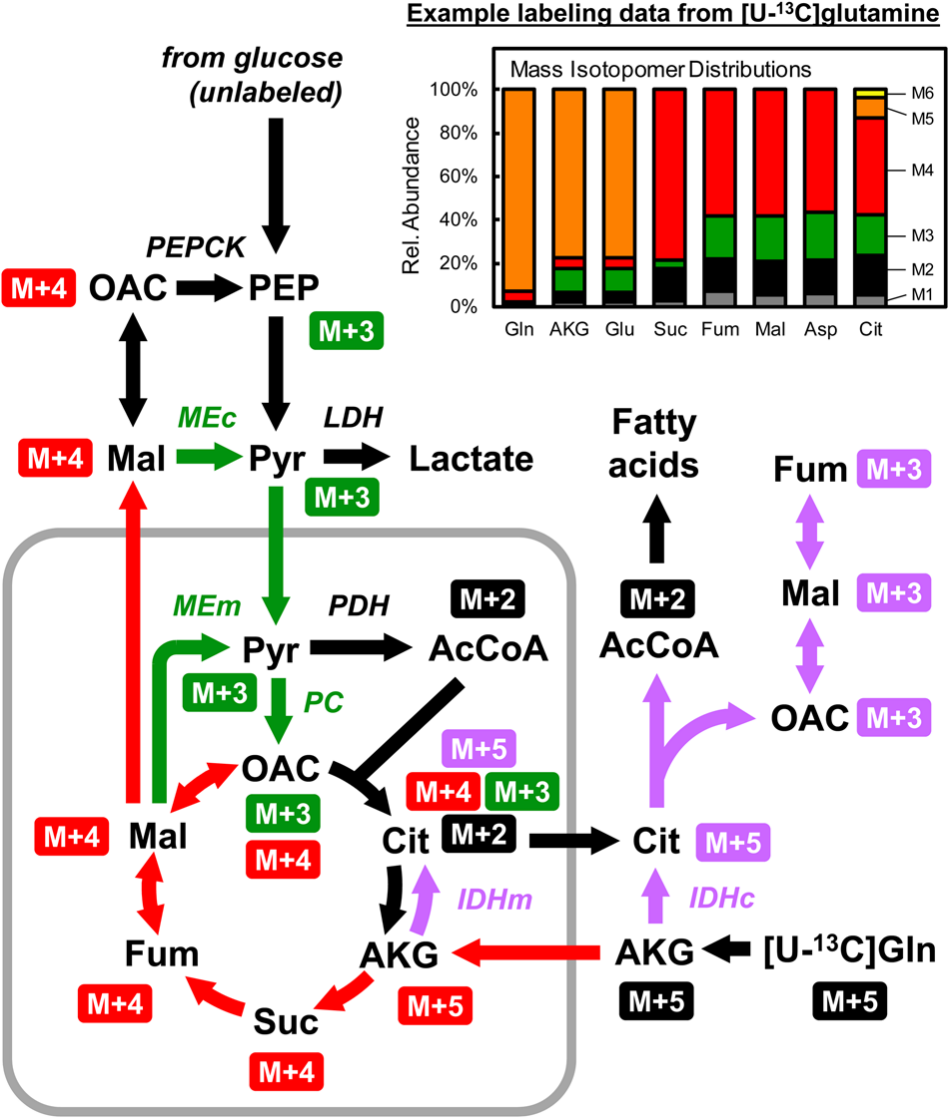
[U-^13^C]Glutamine tracer experiments produce rich labeling patterns in TCA cycle metabolites that allow precise quantification of metabolic fluxes in lower part of central metabolism, i.e., downstream of pyruvate, using ^13^C-MFA. The diagram shows schematically the flow of ^13^C-labeling from [U-^13^C]glutamine into relevant metabolic pathways in cancer cells. The insert shows an example of labeling data obtained from a [U-^13^C]glutamine tracer experiment. Colors of arrows indicate different metabolic pathways: reductive carboxylation of glutamine (purple); glutaminolysis (red); conversion of malate to oxaloacetate via malic enzyme and pyruvate carboxylase (green)

For analysis of fluxes in lower part of central carbon metabolism, i.e., downstream of pyruvate, fully labeled [U-^13^C]glutamine is often used. Glutamine is a the second most highly consumed carbon substrate by many cancer cells (after glucose)^79^; as a result, [U-^13^C]glutamine produces high labeling in metabolites, especially in TCA cycle intermediates, and rich labeling patterns for flux estimation using ^13^C-MFA (Fig. 7). Another advantage of using ^13^C-glutamine as a tracer is that labeling dynamics of ^13^C-glutamine are not affected by the buffering effect of extracellular lactate. Since ^13^C-glutamine labels mainly metabolites downstream of pyruvate, isotopic steady state is reached for the labeled TCA cycle metabolites within a few hours after [U-^13^C]glutamine addition, even when external lactate concentration is high^56^.

In the past decade, [U-^13^C]glutamine tracing has played an important role in elucidating the contribution of glutamine to lipogenesis via reductive carboxylation pathway^13,42^, i.e., via the conversion of glutamine to AKG, then to citrate (i.e., in the reverse direction of TCA cycle, catalyzed by isocitrate dehydrogenases), and finally to AcCoA after cleavage by ATP citrate lyase. To highlight additional flux information that can be obtained from [U-^13^C]glutamine tracer experiments, Fig. 7 shows schematically the flow isotopic labeling from [U-^13^C]glutamine into relevant metabolic pathways. The insert in Fig. 7 shows an example of labeling data set obtained from a [U-^13^C]glutamine tracer experiment. Metabolism of [U-^13^C]glutamine via reductive carboxylation (purple arrows in Fig. 6) results in the production of M + 5-labeled citrate^42^; after cleavage of citrate by ATP citrate lyase, M + 2-labeled AcCoA and M + 3-labeled oxaloacetate are produced (while labeling of oxaloacetate cannot be measured directly, it can be inferred from the labeling of aspartate). In contrast, metabolism of [U-^13^C]glutamine via the glutaminolysis pathway along the normal oxidative direction of the TCA cycle (red arrows in Fig. 6) results in the production of M + 4-labeled succinate, fumarate, malate, and oxaloacetate. M + 4 malate can also produce M + 3-labeled oxaloacetate, after conversion to pyruvate via malic enzyme, followed by carboxylation of pyruvate to oxaloacetate by pyruvate carboxylase (green arrows in Fig. 6). Taken together, [U-^13^C]glutamine tracer experiments produce rich labeling patterns in TCA cycle metabolites that permit precise quantification of metabolic fluxes in these pathways using ^13^C-MFA. In addition to [U-^13^C]glutamine, [5-^13^C]glutamine and [1-^13^C]glutamine have also been used for ^13^C-MFA^13,36,77^. However, in general, these singly labeled glutamine tracers are not as informative as [U-^13^C]glutamine for comprehensive analysis of cellular metabolism.

## Concluding remarks

The isotopic tracing strategies and ^13^C-MFA methods reviewed here present powerful tools for elucidating metabolic flux rewiring in cancer cells. Technically, other stable isotopes such as ^2^H, ^18^O, and ^15^N can also be used to study metabolic phenotypes, and for certain applications these alternative isotope tracers may be preferred^80,81^. From a modeling perspective, the application of multiple isotopes will not cause any problems for MFA. In fact, one of the motivations for developing the EMU framework was to permit and encourage the application of multiple isotopes for flux analysis^39^. Several pioneering studies have already made use of this^45,82^. However, there are several drawbacks and limitations that should be considered when contemplating the use of alternative stable isotopes. For example, ^18^O tracers are generally much more expensive than ^13^C tracers and at present the number commercially available ^18^O tracers is limited. While ^15^N can be used to investigate metabolic pathways where the metabolic intermediates contain N atoms, such as amino-acid pathways, they cannot be used to study central carbon metabolism. Finally, interpretation of ^2^H labeling data is complicated by the presence of significant deuterium kinetic isotope effects. In contrast to ^13^C tracers, where it has been demonstrated that the kinetic isotope effects are negligible^83^, the kinetic isotope effects for ^2^H are substantial^84^. Thus, determining fluxes from ^2^H labeling data is strongly influenced by specific assumptions made regarding the magnitude of kinetic isotope effects for various enzymatic reactions. Still, ^2^H tracers can be valuable in resolving specific aspects of metabolism such as NADPH metabolism in different cellular compartments, which cannot be elucidated with ^13^C tracers^85,86^.

Currently, one of the biggest challenges for ^13^C-MFA in mammalian cells is to resolve compartment-specific fluxes^87^. While certain compartment-specific metabolic fluxes can be determined precisely with ^13^C-MFA, e.g., mitochondrial vs cytosolic malic enzyme fluxes, other fluxes are much more difficult to resolve, e.g., mitochondrial vs cytosolic isocitrate dehydrogenase fluxes. In theory, resolving compartment-specific fluxes would be easier if compartment-specific labeling data could be collected^88^. However, with current protocols for quenching metabolism and extracting intracellular labeling, all intracellular metabolite pools are sampled. As a result, the measured labeling data must be modeled as mixtures from multiple cellular pools^36,61,89^. To resolve compartmentalized metabolism, alternative approaches such as organelle isolation may be valuable in the future^90–92^.

When interpreting ^13^C-MFA results, it is also important to keep in mind that the accuracy of ^13^C-MFA calculations depends strongly on the validity of several modeling assumptions that collectively form the basis for the underlying isotopomer models. These inherent assumptions include the following: (1) metabolic steady-state assumption—it is assumed that metabolic fluxes are constant during the labeling experiment; (2) isotopic steady-state assumption—it is assumed that isotopic labeling does not change in time; (3) no kinetic isotope effect for ^13^C tracers—it is assumed that enzymes cannot discriminate between unlabeled (^12^C) and labeled (^13^C) atoms^83,93^; (4) no metabolite channeling—it is assumed that substrate tunneling via multi-enzyme complexes can be ignored; (5) homogeneous metabolite pools—it is assumed that metabolites within a particular compartment are perfectly mixed; (6) homogeneous cell population—it is assumed that all cells in a culture have the same metabolic phenotype; and (7) no turnover of macromolecules—it is assumed that cellular macromolecules such as proteins, lipids, RNA, and DNA are not broken down and produced at the same time. If one or more of these assumptions are shown to be incorrect for a given biological system, then the ^13^C-MFA methodology must be adjusted to account for these effects. For example, the isotopic ^13^C-NMFA was developed for analysis of systems where labeling data are not constant in time^41,94^, and dynamic MFA methodologies (DMFA and ^13^C-DMFA) were developed for analysis of systems where fluxes are not constant in time^46,95–97^. More recently, the co-culture ^13^C-MFA methodology was developed for analysis of non-homogeneous cell cultures^89^. Turnover of macromolecules such as glycogen, lipids, and RNA has also been observed in many biological systems^98–100^, and these effects can be captured in ^13^C-MFA by adding appropriate dilution fluxes^99^.

Lastly, we want to emphasize the importance of full transparency in reporting ^13^C-MFA results by providing full access to data, models, methods, results, and statistics. As described in this review, ^13^C-MFA results are highly dependent on assumptions and models used for data analysis. As cancer research progresses and new insights are obtained into the unique metabolic features of cancer cells, we may discover additional reactions or pathways that have not been considered before. Reanalyzing past data using updated metabolic models could provide a powerful approach for testing new hypotheses. A recent review paper has proposed minimum data standards to facilitate dissemination of methods, data, and results from ^13^C-MFA studies^44^.
